# Extracorporeal ShockWave Treatment vs. mesotherapy in the treatment of myofascial syndromes: a clinical trial

**DOI:** 10.3389/fmed.2024.1388922

**Published:** 2024-05-22

**Authors:** Dalila Scaturro, Domenico Migliorino, Lorenza Lauricella, Francesco Quartararo, Noemi Calabrese, Sofia Tomasello, Michele Vecchio, Giulia Letizia Mauro

**Affiliations:** ^1^Department of Medicine of Precision in the Medical, Surgical and Critical Care Areas, University of Palermo, Palermo, Italy; ^2^Section of Pharmacology, Department of Biomedical and Biotechnological Sciences, University of Catania, Catania, Italy; ^3^Department of Neuroscience, Biomedicine and Movement of the University of Verona, Verona, Italy

**Keywords:** rehabilitation, musculoskeletal disease, myofascial syndrome, Extracorporeal ShockWave Treatment (ESWT), mesotherapy, trigger points

## Abstract

Numerous scientific papers have compared different treatment options in the management of myofascial pain syndrome. This study evaluated the efficacy of Extracorporeal ShockWave Treatment (ESWT) and mesotherapy in patients with Myofascial Pain Syndrome (MPS) in terms of improvement in pain, functional capacity, and quality of life. A case–control study was conducted on 54 patients, who were randomized into 2 groups: group A, consisting of 27 patients, who were treated with 5 sessions of focal ESWT on a weekly basis; and group B, consisting of 27 patients, who underwent 5 sessions of mesotherapy with Thiocolchicoside fl 4 mg/2 mL and Mepivacaine fl 10 mg/1 mL on a weekly basis. Patients were evaluated at enrollment (T0), after 5 weeks, at the end of rehabilitation treatment (T1), and at a follow- up 30 days after the end of treatment (T2), by administering rating scales (Numeric Rating Scale (NRS) - Pressure Pain Threshold (PPT) – Short Form-36 (SF-36)). The results showed that focal ESWT and Mesoterapy are two valid and effective treatment options in reducing algic symptoms and improving short- and long-term quality of life. However, the use of ESWTs, despite being mildly painful but tolerated, has been shown to be superior to mesotherapy in terms of pain reduction and increased functional capacity.

## 1 Introduction

Myofascial syndrome is a pathology of the musculoskeletal system manifested by referred dull pain associated with functional limitation, contractures, and with possible neuralgic-type manifestations. This condition is characterized by the presence of “trigger points” (TPs) defined as circumscribed focuses of hyper-irritability; the pain caused by acupressure of “TPs” is a “referred” pain, in that it is felt in specific areas (target area), not necessarily adjacent to the stimulated point (1). There are two types of “TPs”: active ones, which are associated with pain even in the absence of movement or palpation; and latent ones, which are painful only upon acupressure; TPs cause muscle weakness and limitation of joint range of motion (2). The pathogenesis of myofascial pain syndrome is still questionable. The most widely accepted hypothesis in the scientific literature attributes the onset of myofascial trigger points (MTrPs) to prolonged contraction of the sarcomere, caused by increased release of acetylcholine at the neuromuscular junction. Pain, therefore, is generated at MTrPs as a result of compression of blood vessels located within the contracted muscle tissue: an ischemic process ensues *in situ* with release of algogenic substances and stimulation of muscle nociceptors (3). In the presence of a constantly altered mechano-metabolic environment, changes occur in the connective tissue constituting the myofascial system, and nociceptive sensation increases: fibroblasts in fact transform into myofibroblasts, contributing to the shortening of the surrounding tissue and increased tone; receptors present within the fascia may transform into nociceptors and become sensitive to mechanical stimuli (allodynia or mechanical hyperalgesia). Therefore, as the structure of connective tissue changes, the polarization of muscle fibers can be altered, thus leading to spontaneous muscle contraction. In addition, at the level of the extracellular matrix, the properties of hyaluronic acid are altered, resulting in increased viscosity and difficulty in the sliding of muscle layers with more difficult muscle contraction (4); nerve endings in the fascia in the more viscous area are stretched, activating constantly and thus generating trigger points. Alterations in blood flow can also cause myofascial pain: the change in flow velocity induces an alteration in the morphology and function of muscle capillaries, causing ischemia during small active movements; this activates type IV nerve endings, contributing to myofascial pain (5). Myofascial syndrome in addition to pain is associated with functional limitation with reduced autonomy in ADLs, leading to alterations in quality of life (6). The diagnosis of myofascial syndrome is made following clinical evaluation by identification of myofascial trigger points: these are sought by palpation of the patient’s painful areas and are defined by the presence of a palpable taut band within the superficial or deep muscle tissue. The muscles involved are usually not uniform, but have heterogeneous areas of different consistencies (7). Myofascial pain syndrome can involve all the muscles of our body, but the muscles of the cervical district (particularly the trapezius muscles, the sternocleidomastoid muscle, the shoulder elevator muscle) are most involved (8, 9), the muscles of the lumbar spine (quadratus lumborum muscle and paravertebral muscles) (10, 11) and the muscles of the lower limbs (tensor fasciae latae muscles, hip adductors, biceps femoris, quadriceps, gastrocnemius and popliteus muscles) (12). Myofascial pain syndrome is often associated with other pathologic conditions of the musculoskeletal system of a chronic degenerative nature that occur in old age such as osteoarthritis (13).

The treatment of myofascial syndrome is varied and heterogeneous; pharmacological therapy with muscle relaxants, antidepressants, weak opioids, local anesthetics, and anti-inflammatories taken orally or topically (in the form of a patch or by mesotherapy) is often used for the management of algic symptoms and painful “PTs” (14, 15). Rehabilitative treatment with active and constant exercise is an effective strategy in the treatment of myofascial pain: it improves joint ROM, mood, and pain threshold, promoting a better quality of life for patients; one of the prescribed exercises is stretching, which stretches myofascial compartments containing PTs and prevents their further occurrence; postural rehabilitation is another rehabilitation option (16). Patients also often undergo massage therapy and manual therapy sessions. Two widely used techniques for the treatment of the aforementioned condition are dry needling and ischemic compressions resulting in increased muscle metabolism (17, 18). Among the most widely used physical therapies in myofascial syndrome are focal ultrasound and shockwaves, which by applying mechanical and thermal energy to the underlying connective tissue, improve circulation, elasticity, and metabolism (19, 20); High Iintensity Laser Therapy (HILT) and Tecartherapy also act on local inflammation, promoting repair of damaged muscle tissue and reducing algic symptoms (21).

Focal shockwaves are acoustic waves (sound pulses, mechanical in nature), characterized by a particular wave shape (first phase of positive pressure, followed by a subsequent rapid, less extensive phase of negative pressure), high energy and short duration, which act on a specific, well-defined point, and therefore are widely used in the treatment of numerous musculoskeletal disorders. They have a beneficial pain-relieving and anti- inflammatory effect (22–24). Analgesic mesotherapy is an outpatient treatment involving multiple mesodermal microinjections of active substances, administered through 27G 0.4 × 4 mm needles, at body parts affected by pain and functional limitation. This technique allows a small amount of drug to be used directly on the area to be treated, reducing systemic drug intake (25, 26).

The aim of the study is to compare treatment with focal Extracorporeal ShockWave Treatment (ESWT) and antalgic mesotherapy in patients with myofascial pain syndrome in terms of pain reduction, increased functional capacity, and autonomy in Activities of Daily Living (ADLs) with improved quality of life.

## 2 Materials and methods

### 2.1 Study design

**Figure 1:**
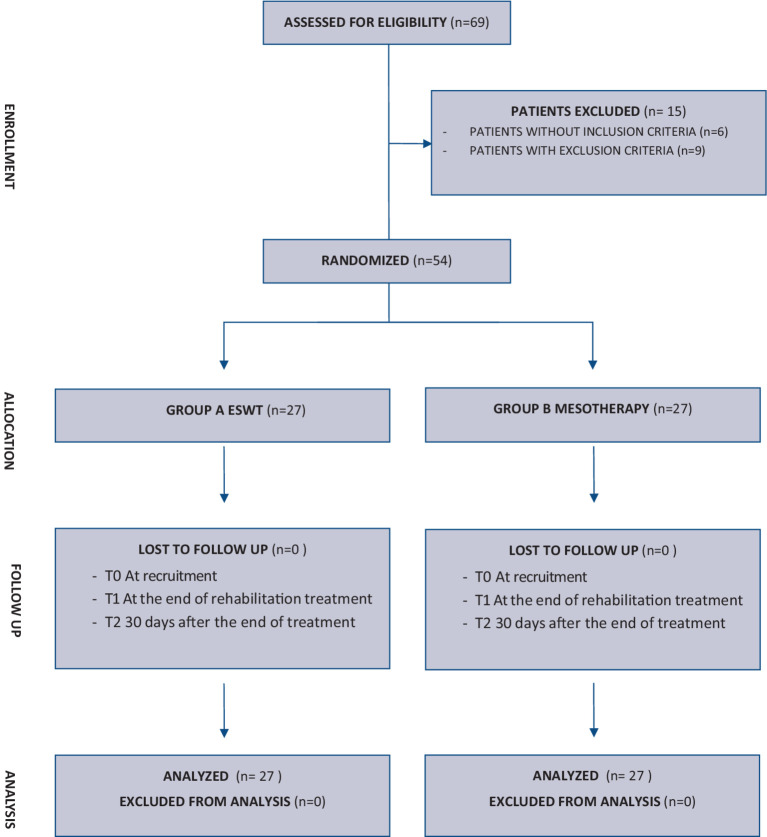
CONSORT 2010 flow diagram.

At the U.O.C. of Functional Recovery and Rehabilitation of the Policlinico “Paolo Giaccone” in Palermo, we conducted a monocenter, unblinded randomized, controlled clinical trial (RTC) on a population of patients with myofascial pain syndrome. The study was conducted between April 2023 and January 2024; for the data collection of this study, we included a consecutive series of patients, who were referred to the U.O.C. of Functional Recovery and Rehabilitation of the A.O.U.P. “Paolo Giaccone” of Palermo during the period between April 2023 and October 2023 to undergo physiatric evaluation. The study received approval from the Local Ethics Committee “Palermo 1” (Approval No. 4/2023) and was conducted following the Declaration of Helsinki. Information and data were processed according to good clinical practice (GCP) guidelines. All subjects signed informed consent before their inclusion, and the study was developed according to CONSORT 2010 Statement: updated guidelines for reporting parallel group randomized trials (Figure 1). The study was registered with ClinicalTrials.gov (NCT06246591).

### 2.2 Participants

The selection criteria were: age 35–65 years, diagnosis of myofascial syndrome (according to the International Association for the Study of Pain - IASP), Numerical Rating Scale (NRS) at T0 ≥ 4, and written informed consent. Patients were excluded from the sample in case of pregnancy, already diagnosed or diagnostically defined neoplasms, coagulation disorders and/or anticoagulant therapy, skin lesions and/or local infections, and contraindications and/or allergies to the active ingredients of mesotherapy. Using our hospital’s database, we enrolled a consecutive series of patients with myofascial pain syndrome who had undergone rehabilitation treatment and met our inclusion criteria. None of the participants dropped out of the study before the scheduled end or experienced any adverse reactions to the proposed treatments.

### 2.3 Intervention

We recruited a total of 54 patients with myofascial syndrome, who were randomly divided into two groups through a system of computer-generated random numbers: group A, consisting of 27 patients, who were treated with 5 sessions of focal ESWT weekly; and group B, consisting of 27 patients, who underwent 5 sessions of mesotherapy with Thiocolchicoside fl 4 mg/2 mL and Mepivacaine fl 10 mg/1 mL weekly.

#### 2.3.1 A group (ESWT)

Group A participants were invited to come to our department’s outpatient clinics, wearing comfortable clothing. Meetings were held weekly, for a total of 5 sessions (5 weeks) lasting about 20 min each. Treatment energy and frequency were established following the recommendations and guidelines of the International Society for Medical Shockwave Treatment (ISMST); specifically, patients underwent focal ESWT (80–100 mJ with 2,250 pulses of 5–10 Hz). Each session involved a 1:1 ratio of patient to physiatrist. The treatment modality was explained to the patient preliminarily, and before each session, the patient was evaluated for trigger points to be treated. Once the TPs were identified, patients were asked to assume a comfortable position, and treatment with focal ESWTs was started as per the ISMST protocol (27).

#### 2.3.2 B group (mesotherapy)

The 27 patients in group B, on the other hand, underwent mesotherapy treatment with Thiocolchicoside fl 4 mg/2 mL and Mepivacaine fl 10 mg/1 mL at our outpatient clinics, once a week, for a total of 5 sessions (5 weeks), lasting about 15 min each. SIM (Italian Mesotherapy Society) standards of good practice were followed (28, 29). Each patient was evaluated before treatment for PTs; after disinfection with Chlorhexidine 2% and sterile gauze, a centrally acting muscle relaxant, Thiocolchicoside fl 4 mg/2 mL, and a local anesthetic, Mepivacaine fl 10 mg/1 mL, diluted in 0.9% NaCl saline, for a final volume of 10 mL were inoculated mesodermally; 6 to 12 microinjections were performed with a 27G 0.4 × 4 mm needle. At the end of the procedure, the patient was monitored for approximately 3 min to exclude adverse reactions or lipothymic episodes.

### 2.4 Clinical evaluation

Demographic and clinical information was obtained from the medical records of the recruited patients. Scores of scales such as the Numerical Rating Scale (NRS) and Pressure Pain Threshold (PPT), to assess the extent of pain; and the Short Form −36 questionnaire (SF-36), which assesses patients’ quality of life, taking into account the individual’s subjective perception regarding health concepts related to activity levels and well-being, were also considered. All of this information was assessed at 3 stages: at enrollment (T0), after 5 weeks, at the end of rehabilitation treatment (T1), and at a follow-up 30 days after the end of treatment (T2). The NRS scale is a one-dimensional 11-point scale that assesses pain intensity in adults, including chronic pain conditions, due to rheumatic diseases. The scale consists of a horizontal line, with a range from 0 to 10, corresponding to “no pain” and “worst pain imaginable,” respectively. The patient indicates the intensity of his or her pain verbally or by drawing a circle on the number that best describes it (30).

The PPT Scale is a means of measuring, documenting, and communicating pressure pain threshold in patients with Myofascial Pain Syndrome and Fibromyalgia, at the level of trigger points and tender points, respectively. It is a 5-point graded scale that includes corresponding criteria for each level. Grade 0 corresponds to no pain or discomfort upon pressure; Grade I mild pain with reported tension without pressure causing body displacement; Grade II indicates moderate pain with stiffening and withdrawal reaction upon pressure; Grade III is associated with severe pain with signs of severe pain associated with verbal gestures and withdrawal of the body part involved; and finally Grade IV is noxious, intolerable and unbearable pain whereby the patient does not even allow palpation of the specific area (31).

The SF-36 is a self-administered, patient-completed questionnaire designed to quantify health status and measure health-related quality of life. It is easy to use, brief, accurate, and easily reproducible. It is a generic, multidimensional instrument consisting of 36 questions that can be divided into 8 scales that analyze physical functioning, limitations due to physical health or emotional problems, energy and fatigue, emotional well- being, social activities, pain, and the patient’s perception of general health. All the scale items present the same response mode by making use of a Likert scale, but with a score that is variable and weighted for each item; to obtain the final result, each item must then be recoded according to a specific formula, and each of the 8 summed scores is then transformed linearly on a scale from 0 (negative health) to 100 (positive health) to provide a score for each subscale (32).

### 2.5 Statistical analysis

Data collection was done through the use of a spreadsheet (Microsoft Excel, version 16.58). We first calculated the sample size of the study, intending to detect an average difference in the rating scales used between group A (ESWT) and group B (mesotherapy). Through the use of the Shapiro–Wilk test, the normality of the collected data was checked. The text and tables report continuous variables, expressed as means and standard deviations, and categorical variables, expressed as absolute numbers and percentages. For statistical analysis of the data, test–t–t was used to compare the averages among the quantitative variables. Finally, to quantify the statistical significance of the difference of the different variables examined between the two groups, we used repeated-measures ANOVA. R statistical software (R Core Team, 2021) was used to analyze the collected data. Results showing *p* ≤ 0.05 were considered statistically significant.

## 3 Results

From daily outpatient evaluations performed from April 2023 to October 2023 at our Functional Recovery and Rehabilitation Unit, we enrolled 69 patients with myofascial pain syndrome. Of these, 6 patients did not fit the inclusion criteria and another 9 had exclusion criteria instead, so only 54 patients were included in the study. The sample size was 51 with a 99% confidence level and a margin of error of 5%. The participants were randomly divided into two groups of equal number and underwent ESTW therapy (Group A) and mesotherapy treatment (Group B).

The demographic characteristics of the sample and initial assessment are summarized in Table 1, which shows the homogeneity of the two groups. The included patients had a mean age of 48.76 ± 9.54 years and included 24 men (44.4 percent) and 30 women (55.6 percent). The sites involved were cervical spine (63%), lumbar spine (18%t), and shoulder muscles (19%). The patients at recruitment had a mean NRS value of 7.03 ± 0.88, a mean PPT Scale of 2.18 ± 0.73. Finally, they had an SF-36 value of 82.5 ± 7.91. No statistically significant between- group differences in baseline characteristics were reported. No statistically significant between-group difference in baseline characteristics was reported (Table 1).

**Table 1:** General characteristics at baseline.

Characteristics	Total (*n* = 54)	Group A (*n* = 27)	Group B (*n* = 27)	*p*-Value
Age mean ± SD	48.76 ± 9.54	49.37 ± 8.95	48.15 ± 10.23	0.64
Sex *n*° %				
Male	24 (44.4%)	11 (40.7%)	13 (48.1%)	
Female	30 (55.6%)	16 (59.3%)	14 (51.9%)	
Localization	Cervical spine (63%)	Cervical spine (58%)	Cervical spine (60%)	
	Lumbar spine (19%)	Lumbar spine (22%)	Lumbar spine (24%)	
	Shoulder (18%)	Shoulder (20%)	Shoulder (16%)	
NRS mean ± SD	7.03 ± 0.88	7 ± 0.92	7.07 ± 0.87	0.77
PPT Scale mean ± SD	2.18 ± 0.73	2,29 ± 0.66	2.07 ± 0.78	0.27
SF-36 mean ± SD	82.5 ± 7.91	81 ± 7.76	84 ± 7.92	0.16

NRS, Numerical Evaluation Scale; PPT, Pressure pain threshold; SF-36, Short Form Health Survey 36.

Table 2 shows the results obtained in group A (ESWT) at T1 and T2. Statistically significant results were found at T1 for all variables considered, with a modest improvement in the values of NRS (4.11 ± 1.12; ≤ 0.05) and PPT Scale (1.18 ± 0.39; ≤ 0.05), as well as SF-36 (93.59 ± 4.54, ≤ 0.05). These results were maintained at T2, but no statistically significant values emerged in terms of pain reduction and improvement in quality of life and autonomy in ADLs (Table 2).

**Table 2:** Effect of treatment with focal ESWT in the A group.

Characteristics	T0	T1	*p*-value	T2	*p*-value
NRS mean ± SD	7 ± 0.92	4.11 ± 1.12	≤ 0.05	3.81 ± 0.96	0.29
PPT Scale mean ± SD	2.29 ± 0.66	1.18 ± 0.39	≤ 0.05	1.07 ± 0.26	0.23
SF-36 mean ± SD	81 ± 7.76	93.59 ± 4.54	≤ 0.05	94.85 ± 3.51	0.26

NRS, Numerical Evaluation Scale; PPT, Pressure pain threshold; SF-36, Short Form Health Survey 36.

Table 3 shows the effects of mesotherapy in group B, at the end of treatment (T1) and 30 days after the end of therapy (T2). In this group, too, there were statistically significant improvements for all research domains at T1: NRS (5.29 ± 1.17; ≤ 0.05), PPT Scale (1.44 ± 0.5; ≤ 0.05) and SF-36 (90.4 ± 5.19; ≤ 0.05); however, again at follow-up 30 days after the end of therapy (T2), no statistically significant values emerged, as shown in the table (Table 3).

**Table 3:** Effect of treatment with mesotherapy in the B group.

Characteristics	T0	T1	*p*-value	T2	*p*-value
NRS mean ± SD	7.07 ± 0.87	5.29 ± 1.17	≤ 0.05	5.11 ± 1.05	0.55
PPT Scale mean ± SD	2.07 ± 0.78	1.44 ± 0.5	≤ 0.05	1.25 ± 0.44	0.14
SF-36 mean ± SD	84 ± 7.92	90.4 ± 5.19	≤ 0.05	91.88 ± 5.22	0.30

NRS, Numerical Evaluation Scale; PPT, Pressure pain threshold; SF-36, Short Form Health Survey 36.

Finally, we compared the results obtained in Group A and Group B at T1. The comparative analysis of the results obtained in the two groups showed substantial differences: in fact, the comparison of the results obtained in the two groups at T1 shows that the patients treated with ESWT (Group A) obtained better results, compared to the patients treated with mesotherapy (Group B) with statistical significance, in terms of pain reduction, assessed by the NRS scale (4.1 ± 1.1 vs. 5.3 ± 1.2; ≤ 0. 05) and the PPT scale (1.2 ± 0.4 vs. 1.4 ± 0.5; ≤ 0.05), and improvement in quality of life, by scores obtained with the SF-36 (93.6 ± 4.5 vs. 90.4 ± 5.2; ≤ 0.05) (Table 4).

**Table 4:** Comparison between the ESWT (Group A) and mesotherapy (Group B) at T1.

Characteristics	Group A	Group B	*p*-value
NRS mean ± SD	4.1 ± 1.1	5.3 ± 1.2	≤ 0.05
PPT Scale mean ± SD	1.2 ± 0.4	1.4 ± 0.5	≤ 0.05
SF-36 mean ± SD	93.6 ± 4.5	90.4 ± 5.2	≤ 0.05

NRS, Numerical Evaluation Scale; PPT, Pressure pain threshold; SF-36, Short Form Health Survey 36.

## 4 Discussion

Myofascial syndrome is a musculoskeletal disorder that is characterized by the presence of “trigger points” (1). In this study, we compared the effects and benefits of two different therapeutic approaches in the management of MPS in terms of improving functional capacity. We also compared the effects of these methods on pain reduction, pain pressure perception, and quality of life. Our results showed that focal ESWT and mesotherapy are two valuable therapeutic proposals in the management of patients with myofascial pain syndrome. In fact, the treatment of myofascial syndrome is multimodal; we make use of pharmacological therapy (anti- inflammatories, muscle relaxants, local anesthetics, antidepressants and weak opioids), (14, 15) often in association with rehabilitative treatment with active and constant exercise, stretching exercises and postural rehabilitation (16).

The synergism of pharmacological therapy and rehabilitative treatment, in association with physical therapies (ESWT-TECARtherapy-HILT) seems to be the best treatment strategy to date (19–20–21). The scientific literature unevenly addresses treatment for myofascial syndrome; in fact, different authors have implemented different treatment options. Ahi et al. (33) compared the effectiveness of high-intensity laser therapy (HILT) and “dry needling” in patients with myofascial pain syndrome and showed that these therapeutic alternatives in addition to exercises contribute to pain reduction. Appasamy et al. (34), on the other hand, conducted a study evaluating the various injection therapies at the level of trigger points in patients with MPS; they compared both the dry needling technique and the various pharmacological administrations of local anesthetics or corticosteroids and concluded that through a detailed history and a proper objective examination, a proper treatment strategy can be developed, appropriate to each patient’s clinical condition.

Several studies have evaluated the effectiveness of dry needling in myofascial pain syndrome, defining its importance in addition to rehabilitative exercises (35), and evaluating verbal suggestion on pain perception during therapy sessions (36, 37). Other popular treatments include manual therapy and dry cupping. Numerous studies have compared the effectiveness of each treatment in short-term relief of myofascial pain (38–42). An additional therapeutic alternative is acupuncture; in the scientific literature many authors have highlighted the effectiveness of this treatment in patients with myofascial pain syndrome (43, 44). Several studies have been performed comparing ESWT with other treatment options: Paoletta et al. (45) performed a review on PubMed to analyze the efficacy of ESWT in patients with myofascial syndrome and found a beneficial role of ESWT in improving clinical and functional outcomes; Yalçın et al. (46) compared the efficacy of kinesiotaping (KT) and ESWT on pain threshold and particularly on coordinated movements by neck muscles and concluded that the combination of exercise, KT and ESWT in MPS was effective in all parameters examined. Nahomi Kuroda et al. (47) also conducted a study comparing “ischemic compressions” (IC) with ESWT demonstrating the effectiveness of ESWT in reducing symptoms. Jun et al. on the other hand, wanted to evaluate the effectiveness of ESWT therapy in myofascial pain syndrome involving neck and shoulder muscles; they stated that ESWT therapy is superior to other treatments in terms of alleviating pain intensity and pressor pain threshold (48). Finally, Hong et al. (49) analyzed different treatments on trigger points at the level of the muscles of the lumbar spine; comparing the treatments, they showed that ESWT was more effective than TPI in relieving pain. In light of the above and with regard to our results, we can state that a study comparing ESWT and antalgic mesotherapy has never been conducted; other strengths of this study are adequate number of samples in both arms and multiple rating scales along with different statistical methods used. The main limitation of our study is the small sample size, therefore, further research should focus on a larger number of patients.

## 5 Conclusion

In patients with myofascial pain syndrome, the use of focal ESWTs can be considered a safe and effective treatment in reducing algic symptoms and improving short- and long-term quality of life. Mesotherapy has also been shown to be an effective alternative in the management of pain in MPS patients; however, ESWT, despite being mildly painful but tolerated, has been shown to be superior to mesotherapy in terms of pain reduction and increased functional capacity, and has not exposed patients to drug intake, albeit by the mesodermal route. It would be desirable in the not-so-distant future to compare the different efficacy of the two treatments for the two different groups on a larger sample of patients.

## Data availability statement

The raw data supporting the conclusions of this article will be made available by the authors, without undue reservation.

## Author contributions

DS: Conceptualization, Data curation, Investigation, Software, Validation, Visualization, Writing – review & editing. DM: Conceptualization, Formal analysis, Investigation, Methodology, Resources, Software, Writing – original draft. LL: Data curation, Validation, Writing – review & editing. FQ: Formal analysis, Investigation, Writing – original draft. NC: Formal analysis, Investigation, Writing – review & editing. ST: Formal analysis, Writing – review & editing. MV: Writing – review & editing. GM: Supervision, Writing – review & editing.
